# C3 glomerulonephritis associated with ANCA positivity: a case report

**DOI:** 10.1186/s12882-021-02354-6

**Published:** 2021-04-21

**Authors:** Ling Li, Li-qin Liu, Ying-ying Yang, Zhang-Xue Hu

**Affiliations:** 1 Renal Division, Department of Medicine, West China Hospital of Sichuan University, 610041 Chengdu, China; 2 West China School of Medicine, Sichuan University, 610041 Chengdu, China

**Keywords:** C3 glomerulonephritis, Membranous nephropathy, Crescent, Renal dysfunction, Anti‐neutrophil cytoplasmic antibody

## Abstract

**Background:**

C3 glomerulopathy (C3G) is a recent disease classification that is characterized by the presence of glomerular deposits (composed of C3) in the absence of significant amounts of immunoglobulin and comprises dense deposit disease and C3 glomerulonephritis (C3GN). Most C3GN manifests as membranoproliferative, mesangial proliferative glomerulonephritis patterns via light microscopy. Pure membranous nephropathy (MN)-like glomerular lesions are rare manifestations of C3GN. Anti-neutrophil cytoplasmic antibodies (ANCAs) are also seldomly reported to be positive in C3GN. Herein, we report the case of a C3GN patient presenting with an MN-like glomerular pattern with ANCA positivity.

**Case presentation:**

A 68-year-old woman was admitted to a local hospital with elevated serum creatinine for two weeks. Laboratory tests showed a hemoglobin level of 85 g/L. Urinalysis was positive for 2 + protein and 360 RBCs/HPF. Blood biochemistry analysis revealed the following concentrations: albumin, 30.3 g/L; globulin, 46.2 g/L; blood urea nitrogen, 19.9 mmol/L; and serum creatinine, 234 µmol/L. The serum C3 level was 0.4950 g/L, and the serum C4 level was 0.1050 g/L. The direct Coombs test was positive. Serologic testing for ANCA revealed the presence of p-ANCA (1:10) by indirect immunofluorescence microscopy assay, as well as the presence of PR3 1.2 (normal range < 1) and MPO 3.5 (normal range < 1) by enzyme immunoassay. Renal biopsy sample pathology showed 2/6 cellular crescents and thickened glomerular basement membranes. Immunofluorescence testing revealed only diffuse, finely granular depositions of C3 along the glomerular capillary walls in frozen and paraffin-embedded tissue sections. Electron microscopy demonstrated the presence of subepithelial electron-dense deposits, similar to those that are observed in membranous nephropathy. Corticosteroid and cyclophosphamide were administered, with a subsequent improvement in renal function.

**Conclusions:**

We present the rare case of a patient with MN-like C3GN with ANCA positivity. C3GN with ANCA positivity may be represented by more crescents, severe renal dysfunction and more extrarenal manifestations. More cases are needed to elucidate the clinicopathologic features and optimal treatments of these patients.

## Background

C3 glomerulopathy (C3G) is a recent disease classification that is characterized by the presence of glomerular deposits (composed of C3) in the absence of significant amounts of immunoglobulin (Ig) [1, 2]. C3G results from the dysregulation of the alternative complement pathway, which may be caused by an acquired or genetic dysfunction of complement regulating proteins [3]. C3G comprises dense deposit disease (DDD) and C3 glomerulonephritis (C3GN), which differ in their appearances upon electron microscopy. The appearance of intramembranous electron-dense deposits (corresponding to the C3 deposits) is characteristic of DDD. Most C3GN manifests as membranoproliferative, mesangial proliferative glomerulonephritis patterns in light microscopy. Electron microscopy shows nondense, intramembranous, mesangial, subendothelial or subepithelial deposits of C3 in C3GN [2, 4]. Isolated subepithelial deposits of C3 are rarely reported.

Anti-neutrophil cytoplasmic antibodies (ANCAs) have been proven to cause pauci-immune necrotizing and crescentic GN and vasculitis [5]. ANCA has become the serologic biomarker for these disorders, with the test having good sensitivity [6]. ANCA can be detected in 25 % of patients with anti-GBM crescentic GN or idiopathic immune-complex crescentic GN [7]. Patients with concurrent ANCA and anti-GBM antibodies have a worse prognosis than that of patients with only ANCA [8, 9]. Until now, only two C3GN patients with ANCA positivity have been reported [10, 11]. Thus, the role of ANCA in C3G patients remains unknown. Herein, we report the rare case a patient with C3GN presenting with isolated subepithelial C3 deposits, cellular crescents and ANCA positivity. The intrinsic mechanism of these symptoms are discussed and detailed.

## Case presentation

A 68-year-old Chinese woman was admitted with elevated serum creatinine for two weeks. She noticed lower back pain and went to a local hospital two weeks prior. There were no symptoms of gross hematuria, foamy urine, frequent urination, urination urgency, urination pain, chills or fever. The laboratory tests revealed a serum creatinine concentration of 374 µmol/L and a urine protein level of 1.23 g/24 h. Therefore, she was transferred to our hospital. No special medication was previously taken by the patient.

Physical examination showed prominent features of facial pallor. Her blood pressure was 143/76 mmHg. There were no palpable lymph nodes. The results of chest and abdominal exams were within normal limits, and mild edema of the lower extremities was noticed.

Laboratory tests showed a hemoglobin concentration of 85 g/L, a white blood cell count of 12.21 × 10^9^/L and a platelet count of 237 × 10^9^/L. Urinalysis was positive for 2 + protein and 360 RBCs/HPF. The urinary protein/creatinine ratio was 0.482 g/mmol Cr. Fecal occult blood test results were negative. The blood biochemistry analysis revealed the following concentrations: albumin, 30.3 g/L; globulin, 46.2 g/L; blood urea nitrogen, 19.9 mmol/L; serum creatinine, 234 µmol/L; and uric acid, 254 µmol/L. Procalcitonin and C-reactive protein results were negative. The alpha fetoprotein, carbohydrate antigen-CA125, carbohydrate antigen-CA199 and carcinoembryonic antigen results were negative. Anti-nuclear antibody (ANA), anti-dsDNA and anti-Sm antibody results were negative. The serum C3 concentration was 0.4950 g/L (normal range: 0.785–1.520 g/L), and serum C4 concentration was 0.1050 g/L (normal range: 0.145–0.360 g/L). The direct Coombs test was positive. Serologic testing for ANCA revealed the presence of p-ANCA (1:10) by indirect immunofluorescence microscopy assay (IFA), and PR3 1.2 (normal range < 1) and MPO 3.5 (normal range < 1) by enzyme immunoassay (EIA). The serum IgG concentration was 36.0 g/L (normal range: 8-15.5), whereas the serum IgG4 concentration was 1.19 g/L. Immunofixation electrophoresis did not reveal the presence of monoclonal immunoglobulin. Renal ultrasonography showed that the size of the right kidney was 97 × 46 × 45 mm^3^, whereas the left kidney measured 98 × 41 × 45 mm^3^.

**Fig. 1:**
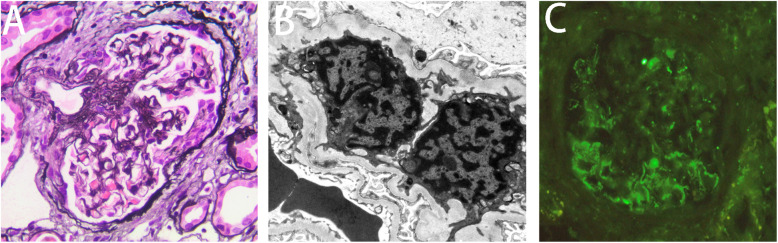
The histopathologic findings of the renal specimen. Panel **a** shows a cellular crescent in a glomerulus (periodic acid-silver methenamine staining, ×400); Panel **b** shows subepithelial electron-dense deposits (x15000); Panel **c** shows dominant granular C3 staining along the glomerular capillary wall (frozen sample, immunofluorescence, ×400)

A renal biopsy was performed. Light microscopy demonstrated the presence of four nearly normal glomeruli, except for the presence of two cellular crescents (Fig. 1 a). The glomerular basement membrane (GBM) appeared to be thickened. Interstitial inflammatory cells infiltration was also observed. The infiltrating inflammatory cells predominantly consisted of plasma cells, lymphocytes and eosinophils. No arteriole necrosis was observed. Immunofluorescence testing was negative for IgG, IgM, IgA, C4, C1q, κ and λ in the glomeruli of frozen tissue sections. Diffuse, finely granular deposits of C3 along the glomerular capillary walls were observed (Fig. 1b). The results were the same in the paraffin-embedded tissue sections after antigen retrieval. Electron microscopy demonstrated subepithelial electron-dense deposits, similar to those observed in MN, which seemed to erode the GBM and make it extremely slim and vulnerable to breakage (Fig. 1 c). No subendothelial or mesangial electron-dense deposits were observed. On this basis, the patient was diagnosed with C3GN with ANCA positivity. Further analysis of the complement components and genetic mutations was not performed because of the patient`s refusal.

**Fig. 2:**
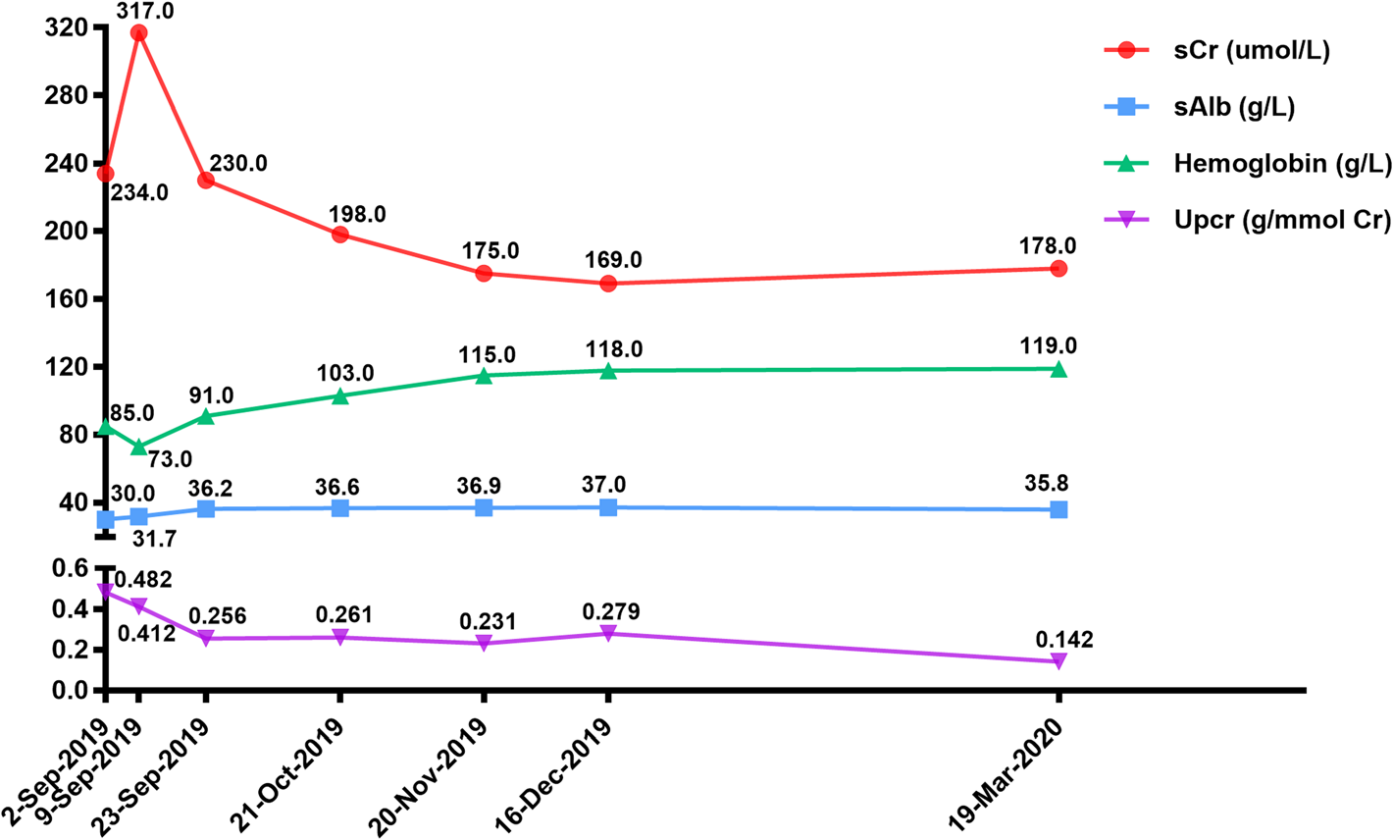
The clinical course of the patient pre- and post treatment

She was treated with 500 mg intravenous methylprednisolone for 3 days, followed by 30 mg oral prednisone per day, which was slowly tapered. Moreover, 400 mg intravenous cyclophosphamide was prescribed to be taken once a month. After treatment for six months, the serum creatinine level decreased to 178.0 µmol/L, and the serum albumin increased to 34.8 g/L (Fig. 2). Hemoglobin increased to 119 g/L. The urinary protein/creatinine ratio decreased to 0.142 g/mmol. The serum C3 level was 0.62 g/L, and the IgG level was 9.3 g/L. The ANCA was p-ANCA (1:10), whereas PR3 and MPO tests were negative.

## Discussion and conclusions

C3G is caused by the abnormal activation of complements due to the dysregulation of the alternative complement pathway. C3GN is a subtype of C3G, and it is characterized by dominant, nondense C3 deposits in the glomeruli, with an intensity of two or more orders of magnitude greater than any other Ig (0–3 scale). C3GN can affect people of all ages. The average age of onset is approximately 30 years. The presentations of C3GN are heterogeneous and include proteinuria (mild to nephrotic syndrome), microhematuria, hypertension and chronic renal failure [12]. Moreover, 25 % of C3GN patients progress to end-stage renal disease in ten years. C3GN recurs in 60 % of allografts. Approximately 40 % of C3GN patients present with decreased serum C3 levels. C3 nephritic factor (C3NeF) is detectable in 45 % of patients. Variants of factor H, factor I, membrane co-factor protein and the complement factor H-related protein family (CFHR1-5) can be detected. The majority of patients with C3GN manifest MPGN, followed by diffuse proliferative GN, crescentic GN and mesangial proliferative GN [12, 13]. Isolated subepithelial deposits are rare [12, 14]. Paraffin IF staining is used to identify monoclonal immunoglobulin glomerular deposits to avoid the misdiagnosis of Ig-mediated glomerulonephritis as C3GN [15]. In this case, subepithelial C3 deposits without Ig were confirmed by frozen and paraffin IF staining. When combined with the features of electron microscopy (Fig. 1 c), the patient was diagnosed as having C3GN with MN-like patterns. This case presents a rare pathologic subtype of C3GN and highlights the importance of EM and paraffin IF staining in differentiating MN-like patterns from true membranous nephropathy.

It is noticeable that this patient exhibited serologic ANCA-positive results via IIF and ELISA. ANCA is strongly associated with small-vessel, pauci-immune vasculitis, such as granulomatosis with polyangiitis, microscopic polyangiitis and eosinophilic granulomatosis with polyangiitis [16]. ANCA testing has a good sensitivity for the detection of small-vessel pauci-immune vasculitis. Complement activation, predominantly via the alternative pathway [17, 18], serves an important role in the pathogenesis of ANCA-associated glomerulonephritis (ANCA-GN) [19–21]. C3 was positive in 41.7 % of patients with ANCA-associated glomerulonephritis via immunohistochemistry. However, electron-dense deposits were detected in only 3 of 47 patients. In renal biopsy samples from these three patients, electron-dense deposits were small in size and mesangially located. Mild immunoglobulin deposits were also found in these three patients [18]. These results suggest that C3-dominant electron-dense deposits are rare in ANCA-GN. Therefore, in our case, the C3 deposits should not have been caused by ANCA because of the dominant subepithelial electron-dense deposits.

Until now, only two C3GN cases with ANCA positivity have been reported. Chaudhuri reported the case of a 75-year-old male presenting with acute kidney injury with high levels of serum serine protease 3 antibodies (C-ANCAs) and rheumatoid factor (132 IU/mL), low serum C3 levels (57 mg/dL), severe anemia and thrombocytopenia [10]. A renal biopsy sample revealed subendothelial and mesangial C3 deposits, with half of the glomeruli being globally sclerotic, and the presence of small crescents in the nonsclerotic glomeruli. Prednisone and cyclophosphamide were administered, with a subsequent improvement in renal function and the discontinuation of hemodialysis. Jianan Feng reported the case of a 72-year-old male who presented with fever, anemia, elevated IgG4 (7.23 g/L), a high erythrocyte sedimentation rate, and MPO-ANCA positivity (p-ANCA) [11]. The serum complement C3 level was normal. A renal pathologic study revealed the presence of cellular crescents in half of the glomeruli, as well as the presence of subepithelial and mesangial C3 deposits. Diffuse IgG4-positive inflammatory cell infiltration was also noticeable. This patient was diagnosed as having ANCA-associated vasculitis, IgG4-associated tubulointerstitial nephritis and C3GN. The patient’s condition improved with a treatment of methylprednisolone and cyclophosphamide. Singh reported the case of a 10-year-old DDD patient presenting with acute kidney injury, nonpruritic generalized skin rash, hip pain and fever. ANCA results were negative. A renal pathologic study showed the presence of crescentic necrotizing GN with isolated C3 deposits and intramembranous and subepithelial dense deposits, which mimicked renal small-vessel vasculitis [22]. Our patient showed cellular crescents, isolated subepithelial C3 deposits, MPO-ANCA positivity (p-ANCA) and anemia, and the patient’s renal function improved with immunosuppressive treatment. It is noticeable that in these last three cases of C3G (two with C3GN and one with DDD), the patients presented with acute kidney injury, crescents, subepithelial C3 deposits and extrarenal manifestations. Singh theorized that a preexisting mild form of C3 glomerulopathy (C3GN/DDD) without severe hypocomplementemia and GBM remodeling may have been present [22]. Infection may aggravate the complement abnormalities, thus resulting in the deposition of large subepithelial complement-rich deposits, which weaken the paramesangial GBM, causing rupture, crescent formation and the presentation of renal failure. Furthermore, ANCA plays an important role in the pathogenesis of ANCA-associated vasculitis and causes crescentic necrotizing GN. Patients with ANCA-positive lupus nephritis presented with a higher degree of glomerular necrosis, higher dsDNA titers, lower serum C4 concentrations and higher serum creatinine levels at the time of biopsy, as well as a higher chronicity index [23]. ANCA may independently contribute to more vasculitis-like lesions in C3 glomerulopathy, such as glomerular necrosis and crescents. It is interesting that patients with ANCA-positive C3 glomerulopathy often present with extrarenal manifestations, such as fever, hemolytic anemia (due to a positive Coombs test), elevated globulin and skin rash, among other symptoms. ANCA-associated vasculitis involves multiple organs. The activation of complement proteins, whether in ANCA-associated vasculitis or C3GN, can contribute to hemolytic anemia [24].

An optimal disease-directed treatment for C3G has yet to be determined. Angiotensin-converting enzyme inhibitors or angiotensin II receptor blockers can be used for their antiproteinuric effects. Based on the central role of complement abnormalities in the pathogenesis of C3G, anticomplement therapies seem to be potential treatments. Several case reports and small trials have supported the utility of anti-C5 therapy (eculizumab) in C3G patients, especially for patients with elevated soluble C5b-9 levels [25]. Immunosuppression may be beneficial for patients with C3GN by limiting the anaphylatoxic effects of C3a and C5a, thus inhibiting immune cell reactions and inflammation, as well as reducing antibody production [26]. The three ANCA-positive C3GN patients, including our patient, presented with positive ANCA results, cellular crescents and elevated serum creatinine. Treatment with steroids and cyclophosphamide provided beneficial effects in these three patients, with improvements in renal function and extrarenal manifestations. The effect of anti-C5 therapy for this subtype of C3GN remains unknown.

Herein, we presented the rare case of a patient with C3GN with predominant MN-like lesions, crescents and positive ANCA results. Patients with C3GN with ANCA positivity may present with more crescents, more severe renal dysfunction and more extrarenal manifestations. More cases are needed to elucidate the clinicopathologic characteristics and optimal treatment of these patients.

## Data Availability

All generated or analyzed data were obtained from the West China Hospital of Sichuan University, and they are included in this published article.

## Abbreviations

C3G: C3 glomerulopathy
DDD: Dense deposit disease
C3GN: C3 glomerulonephritis
ANCA: Anti-neutrophil cytoplasmic antibodies
MN: Membranous nephropathy
ANA: Anti-nuclear antibody
IFA: Immunofluorescence microscopy assay
EIA: Enzyme immunoassay
IFE: Immunofixation electrophoresis
GBM: Glomerular basement membrane
GN: Glomerulonephritis
